# Alterations and diagnostic performance of capillary ketonemia in pediatric acute appendicitis: a pilot study

**DOI:** 10.1007/s00383-022-05332-7

**Published:** 2022-12-10

**Authors:** Javier Arredondo Montero, Mónica Bronte Anaut, Carlos Bardají Pascual, Giuseppa Antona, Natalia López-Andrés, Nerea Martín-Calvo

**Affiliations:** 1 Pediatric Surgery Department, Hospital Universitario de Navarra, Calle Irunlarrea 3, 31008 Pamplona, Navarra Spain; 2 School of Medicine, University of Navarra, Pamplona, Navarra Spain; 3 Pathology Department, Hospital Universitario de Araba, Vitoria, Spain; 4 Cardiovascular Translational Research, NavarraBiomed (Miguel Servet Foundation), Hospital Universitario de Navarra, Universidad Pública de Navarra (UPNA), IdiSNA, Pamplona, Navarra Spain; 5 School of Medicine, Department of Preventive Medicine and Public Health, University of Navarra, Pamplona, Spain; 6 IdiSNA, Instituto de Investigación Sanitaria de Navarra, Pamplona, Spain; 7 CIBER de Fisiopatología de la Obesidad y la Nutrición, Instituto de Salud Carlos III, Madrid, Spain

**Keywords:** Capillary, Ketonemia, Ketone bodies, Dehydratation, Assessment tool, Pediatric acute appendicitis, Diagnostic, Specificity, Sensitivity, ROC, AUC, Youden index, Complicated, Non complicated

## Abstract

**Introduction:**

The diagnostic performance of capillary ketonemia (CK) has been previously evaluated in context of pediatric acute gastroenteritis. To our knowledge, there is no literature on its performance in the setting of pediatric acute appendicitis (PAA).

**Materials and methods:**

In this study, 151 patients were prospectively included and divided into two groups: (1) patients with non-surgical abdominal pain in whom the diagnosis of PAA was excluded (*n* = 53) and (2) patients with a confirmed diagnosis of PAA (*n* = 98). In 80 patients (Group 1, *n* = 23 and group 2, *n* = 57) a CK was measured at the time of diagnosis. The PAA group was further classified into complicated (*n* = 18) and uncomplicated PAA (*n* = 39). Quantitative variables were compared between groups using the Mann–Whitney *U* test. Diagnostic performance of CK was evaluated with ROC curves.

**Results:**

CK values were 0.3 [0.1–0.9] mmol/L in group 1 and 0.7 [0.4–1.4] mmol/L in group 2 (*p* = 0.01). Regarding the type of PAA, CK values were 0.6 [0.4–0.9] mmol/L in uncomplicated PAA and 1.2 [0.8–1.4] mmol/L in complicated PAA (*p* = 0.02). The AUC for the discrimination between groups 1 and 2 was 0.68 (95% IC 0.53–0.82) (*p* = 0.24) and the AUC for the discrimination between uncomplicated PAA and complicated PAA was 0.69 (95% IC 0.54–0.85) (*p* = 0.04). The best cut-off point (group 1 vs group 2) resulted in 0.4 mmol/L, with a sensitivity of 80.7% and a specificity of 52.2%. The best cut-off point (non-complicated vs complicated PAA) resulted in 1.1 mmol/L, with a sensitivity of 61.1% and a specificity of 76.9%.

**Conclusions:**

This study found significantly higher levels of CK in patients with PAA than in those with NSAP. Similarly, significantly higher levels were observed in patients with complicated than in those with uncomplicated PAA. Nevertheless, the diagnostic performance of CK was only moderate in the two settings analyzed. The potential usefulness of CK determination as a tool to guide the preoperative rehydration regimen of patients with PAA to prevent postoperative hyporexia and vomiting is a promising line of research and should be evaluated in future studies.

**Supplementary Information:**

The online version contains supplementary material available at 10.1007/s00383-022-05332-7.

## Introduction

Pediatric acute appendicitis (PAA) is the most frequent abdominal surgical pathology in the world, with a cumulative lifetime risk of up to 8% according to different series [1]. The lack of specificity of the clinical findings and the wide differential diagnosis of pediatric abdominal pain makes this pathology present a significant rate of error and diagnostic delay [2], which entails a significant increase in attributable morbidity and mortality as well as in healthcare costs [3].

In the last decade, several studies evaluated different biomarkers as potential diagnostic tools for PAA, but the results were inconsistent, probably due to small sample sizes and heterogeneity between different series. Recently published systematic reviews contributed to synthesize the existing evidence and guide new lines of research. Interleukin-6, calprotectin and pentraxin-3 constitute illustrative examples of this [4–6]. Likewise, the distinction between complicated and uncomplicated PAA, which is becoming increasingly relevant given the growing consideration of non-surgical management of uncomplicated PAA, is being studied at the expense of this type of diagnostic tools. One example is the lymphocyte-to-monocyte ratio, which has emerged as a useful biomarker to discriminate between complicated and uncomplicated PAA [7].

Most of the studies aimed at optimizing the management of patients with PAA focused on surgical aspects, and the evidence on the potential impact of more conservative measures applied during the preoperative period is scarce. It is known that intravenous rehydration significantly reduces the levels of ketone bodies, which are responsible of the perpetuation of nausea and vomiting [8–10]. Ketone bodies can be determined in capillary blood and in urine. Because capillary beta hydroxybutyrate (β-OHB) has higher sensitivity than urinary ketone, previous studies recommended the use of capillary β-OHB to assess patient’s metabolic status [11]. To our knowledge, there is no literature evaluating the ketosis status of patients with PAA prior to surgery. Therefore, the potential effect that ketone body levels-guided intravenous hydration of patients with PAA prior to surgery could have on recovery time and hospital stay is unknown [12].

In this study, we aimed to compare the level of capillary ketonemia (CK) between patients with PAA and controls and determine whether CK may be a valid tool for the diagnosis of PAA and for the discrimination between complicated and uncomplicated PAA.

## Materials and methods

This study was approved by our center's clinical research ethics committee prior on December 18, 2020, under code PI_2020/112. The ethical principles of the Declaration of Helsinki were applied for the conduct of this research study. The parents or legal representative of all participants signed an informed consent form prior to the inclusion in the study.

### Study design

This is a subanalysis of the BIDIAP study [13, 14], an observational prospective study aimed to assess the diagnostic performance of several serum biomarkers in PAA. The objective of this subanalysis was to characterize the alterations in CK of patients with PAA and calculate its diagnostic performance. Two groups of pediatric patients were included in this study: (1) patients with acute abdominal pain who were admitted at the emergency department in whom there was a diagnostic suspicion of acute appendicitis and in which it was finally excluded—also defined as non-surgical abdominal pain (NSAP), and (2) patients with histologically confirmed diagnosis of acute appendicitis. For further analysis, patients in group 2 were stratified based on histopathological classification of the appendix in uncomplicated PAA (congestive, phlegmonous or suppurative appendicitis) and complicated PAA (gangrenous or perforated appendicitis). All patients in group 1 were contacted 2 weeks after their inclusion in the study to confirm that they had not been diagnosed with PAA in that period.

Sociodemographic and clinical variables were collected at baseline through a paper-based questionnaire completed by parents or legal guardians. Information on analytical, surgical, radiological and histological variables was extracted from participants’ clinical records.

Patients were recruited when the personnel conducting the investigation were available at the center. The recruitment period extended from February to December 2021. Inclusion and exclusion criteria are shown in Supplementary file 1.

### Sample collection and determination of the capillary ketone

In all patients, the blood sample for the determination of CK was taken at the time of inclusion in the study, upon arrival at the pediatric emergency department and before initiating intravenous hydration. Blood sample was extracted by finger prick and placed on a blood beta-ketone test strip (Abbott ©, Abbott Diabetes Care Ltd). The analysis was performed by electrochemical study in a FreeStyle Optimum Neo device.

### Statistical analysis

For descriptive purposes, we used mean and standard deviation or median and interquartile ranges for quantitative variables and proportions for categorical ones. Kolmogorov–Smirnov test was used to assess the normality of quantitative variables. Sociodemographic, clinical and analytical variables were compared between groups using the Mann–Whitney *U* test. To calculate the discriminative capacity of the biomarkers, we calculated the area under the receiver operating characteristic curves (ROC). Statistical significance was settled in a *p*-value < 0.05. Statistical analysis was performed with STATA 17.0 (StataCorp LCC).

We calculated the Youden Index for each value with the following formula:

1 $${\text{Sensitivity}}+{\text{Specificity}}-1$$

The biomarker value with the highest Youden Index was considered the optimal cut-off point.

Pearson’s or Spearman’s correlation tests were used to assess the correlation between CK and both clinical and analytical variables.

## Results

### Sociodemographic and clinical characteristics

This study, part of the BIDIAP cohort [13–15], included 151 patients, divided into two groups: (1) patients with non-surgical abdominal pain in whom the diagnosis of PAA was excluded (*n* = 53) and (2) patients with a confirmed diagnosis of PAA (*n* = 98). In 80 patients (group 1 = 23 patients, group 2 = 57 patients) a capillary ketone blood determination was obtained at the time of diagnosis. In the remaining 71 patients, no such determination was obtained, either because the parents and/or patients expressly refused the capillary finger prick or because of errors in the reading of the test strip.

Given the high drop off rate of the cohort (47%) and to assess the possibility of selection bias we performed a detailed comparative analysis between the two subpopulations of the cohort: patients with capillary ketonemia determination present (*n* = 80) and patients without such determination (*n* = 71). This analysis, which included all the sociodemographic and clinical variables presented in this paper, found no statistically significant differences in any of the analyses performed.

Patients in group 2 (*n* = 57) were further subdivided into complicated PAA (*n* = 18) and uncomplicated PAA (*n* = 39). Sociodemographic characteristics of patients by group are shown in Table 1. Significant differences between groups were observed for height (*p* = 0.05) and weight (*p* < 0.01).

**Table 1:** Sociodemographic characteristics of study participants by group (NSAP vs PAA)

Sociodemographics	Group 1 (NSAP) *N* = 23	Group 2 (PAA) *N* = 57	*P*-value
Age (years)	11.21 (2.64)	9.87 (3.08)	0.08
Sex (Male/Female) (%)	13/10 (56.52%)	39/18 (68.42%)	0.23
Height (centimeters)	1.50 (0.17)	1.41 (0.19)	0.05
Weight (kilograms)	47.27 (16.86)	36.75 (14.74)	< 0.01

Numbers are mean (standard deviation) or number (percentage)

Clinical characteristics of patients by group are shown in Table 2. No significant differences were observed for the time of pain evolution, the prevalence of hyporexia or the presence of fever at home. However, compared with patients with NASP, those with PAA had significantly more emetic episodes (*p* < 0.01). In relation to analytical determinations (Table 2), we found that patients in group 2 had significantly higher levels of total leukocytes (*p* < 0.01), total neutrophils (*p* < 0.01) C- reactive protein (CRP) (*p* < 0.01) and procalcitonin (PCT) (*p* < 0.01).

**Table 2:** Clinical characteristics and analytical values of study participants by group (NSAP vs PAA)

Clinical variables	Group 1 (NSAP) *N* = 23	Group 2 (PAA) *N* = 57	*P*-value
Hours of pain evolution	28.09 (22.99)	26.02 (19.93)	0.85
Fever > 37.8 (Yes/No) (%)	6/17 (26.08%)	18/39 (31.57%)	0.42
Number of diarrheal stools	0.21 (1.04)	0.39 (1.46)	0.39
Urinary symptoms (Yes/No/Missing) (%)	4/19 (17.4%)	14/43 (24.56%)	0.35
Number of emetic episodes	0.22 (0.60)	2.30 (2.28)	< 0.01
Hyporexia (Yes/No) (%)	18/5 (78.26%)	48/9 (81.36%)	0.36
Leucocytes (1 × 10^9/L)	9.83 (3.21)	16.47 (5.13)	< 0.01
Neutrophils (1 × 10^9/L)	6.24 (3.08)	13.36 (5.12)	< 0.01
CRP (mg/L)	21.24 (36.78)	44.96 (49.48)	< 0.01
PCT (ng/mL)	0.09 (0.17)	1.79 (6.98)	< 0.01
Serum creatinine (mg/dL)*	0.63 [0.56–0.68]	0.62 [0.55–0.66]	0.20
Serum sodium (mmol/L)*	138 [137–139]	137 [135–138]	0.08
Capillary ketonemia (mmol/L)*	0.3 [0.1–0.9]	0.7 [0.4–1.4]	0.01

Numbers are mean (standard deviation) or numbers (percentage)

*Median, Interquartile range

Table 3 shows the comparison of clinical variables between complicated and uncomplicated PAA groups. No significant differences were observed for the time of pain evolution, the prevalence of hyporexia or the number of emetic episodes. However, patients with complicated PAA were more likely to have fever at home than those with uncomplicated PAA (*p* = 0.01). The comparison of analytical determinations between the complicated and uncomplicated PAA groups (Table 3) showed significantly higher levels of total leukocytes (*p* < 0.01), total neutrophils (*p* = 0.01), CRP (*p* = 0.02) and PCT (*p* < 0.01) in patients with complicated PAA.

**Table 3:** Clinical characteristics and analytical values of patients by group (complicated vs. uncomplicated PAA)

Clinical variables	Uncomplicated PAA *N* = 39	Complicated PAA *N* = 18	*P*-value
Hours of pain evolution	23.95 (19.43)	30.5 (20.84)	0.11
Fever at home > 37.8 (Yes/No) (%)	8/31 (20.51%)	10/8 (55.55%)	0.01
Number of diarrheal stools	0.38 (1.39)	0.39 (1.65)	0.43
Urinary symptoms (Yes/No) (%)	10/29 (25.64%)	4/14 (22.22%)	0.53
Number of emetic episodes	2.10 (2.27)	2.72 (2.32)	0.29
Hyporexia (Yes/No) (%)	33/6 (84.61%)	15/3 (83.33%)	0.59
Leucocytes (1 × 10^9/L)*	15.31 (4.96)	18.98 (4.67)	< 0.01
Neutrophils (1 × 10^9/L)*	12.22 (5.11)	15.82 (4.32)	0.01
CRP (mg/L)*	33.16 (38.81)	70.55 (60.70)	0.02
PCT (ng/mL)*	1.48 (7.65)	2.45 (5.51)	< 0.01
Serum creatinine (mg/dL)*	0.61 [0.54–0.67]	0.62 [0.58–0.65]	0.66
Serum sodium (mmol/L)*	137 [136–139]	136 [135–138]	0.14
Capillary ketonemia (mmol/L)*	0.6 [0.4–0.9]	1.2 [0.8–1.4]	0.02

Numbers are mean (standard deviation) or numbers (percentage)

*Median, Interquartile range

None of the patients included in this study presented hypoglycemia. No significant differences in serum sodium or creatinine levels were found in either of the two univariate comparative analyses performed.

### Capillary ketonemia

**Fig. 1:**
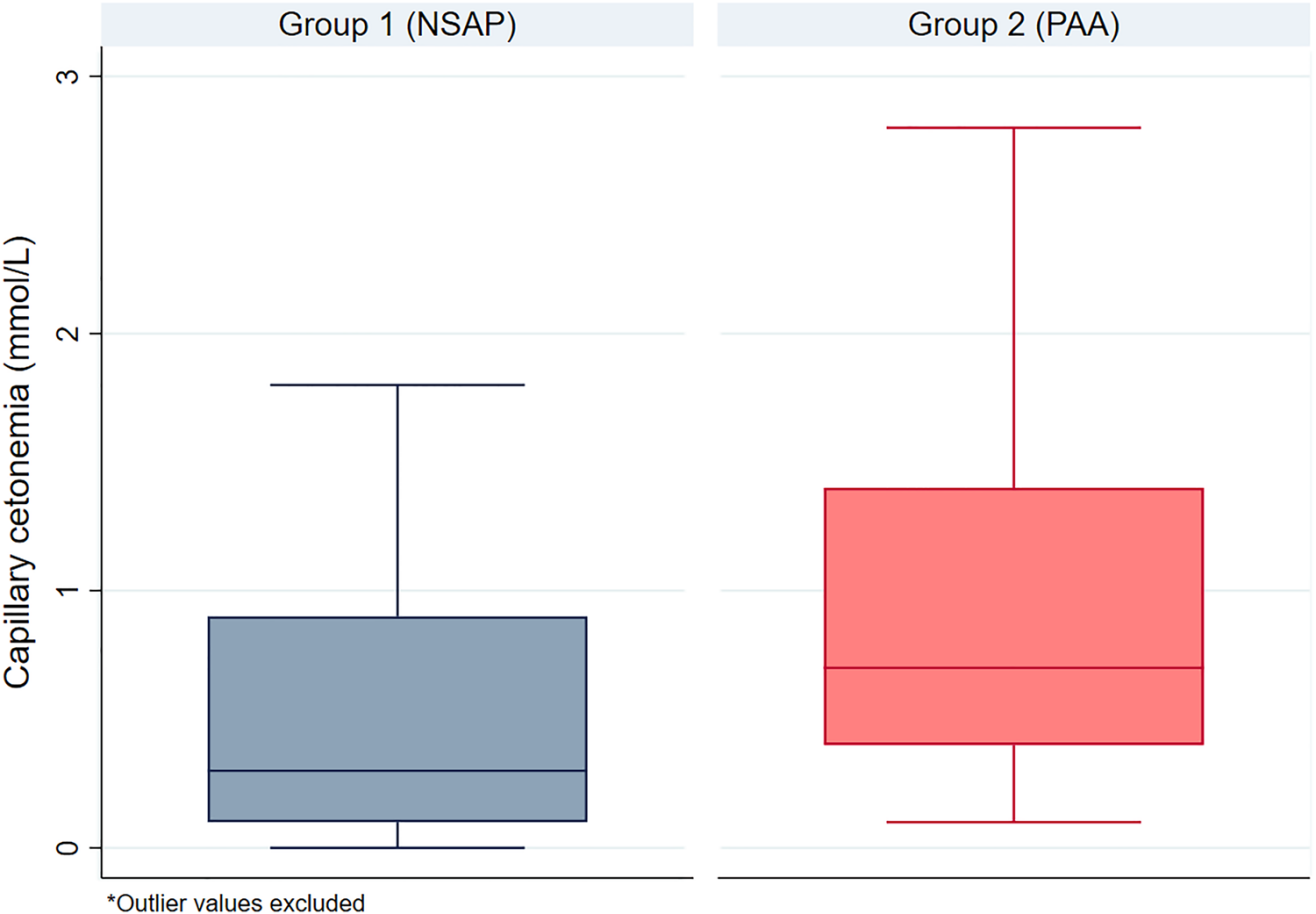
Box-plot representation of Capillary ketonemia values in groups 1 and 2

CK values were 0.3 [0.1–0.9] mmol/L in group 1 and 0.7 [0.4–1.4] mmol/L in group 2 (*p* = 0.01). Regarding the type of PAA, CK values were 0.6 [0.4–0.9] mmol/L in uncomplicated PAA and 1.2 [0.8–1.4] mmol/L in complicated PAA (*p* = 0.02). Figure 1 shows the graphical representation of the determinations of CK in groups 1 and 2 (2 outliers from group 1 and 2 outliers from group 2 were removed for a better visualization of data distribution).

### Diagnostic performance of CK in PAA

**Fig. 2:**
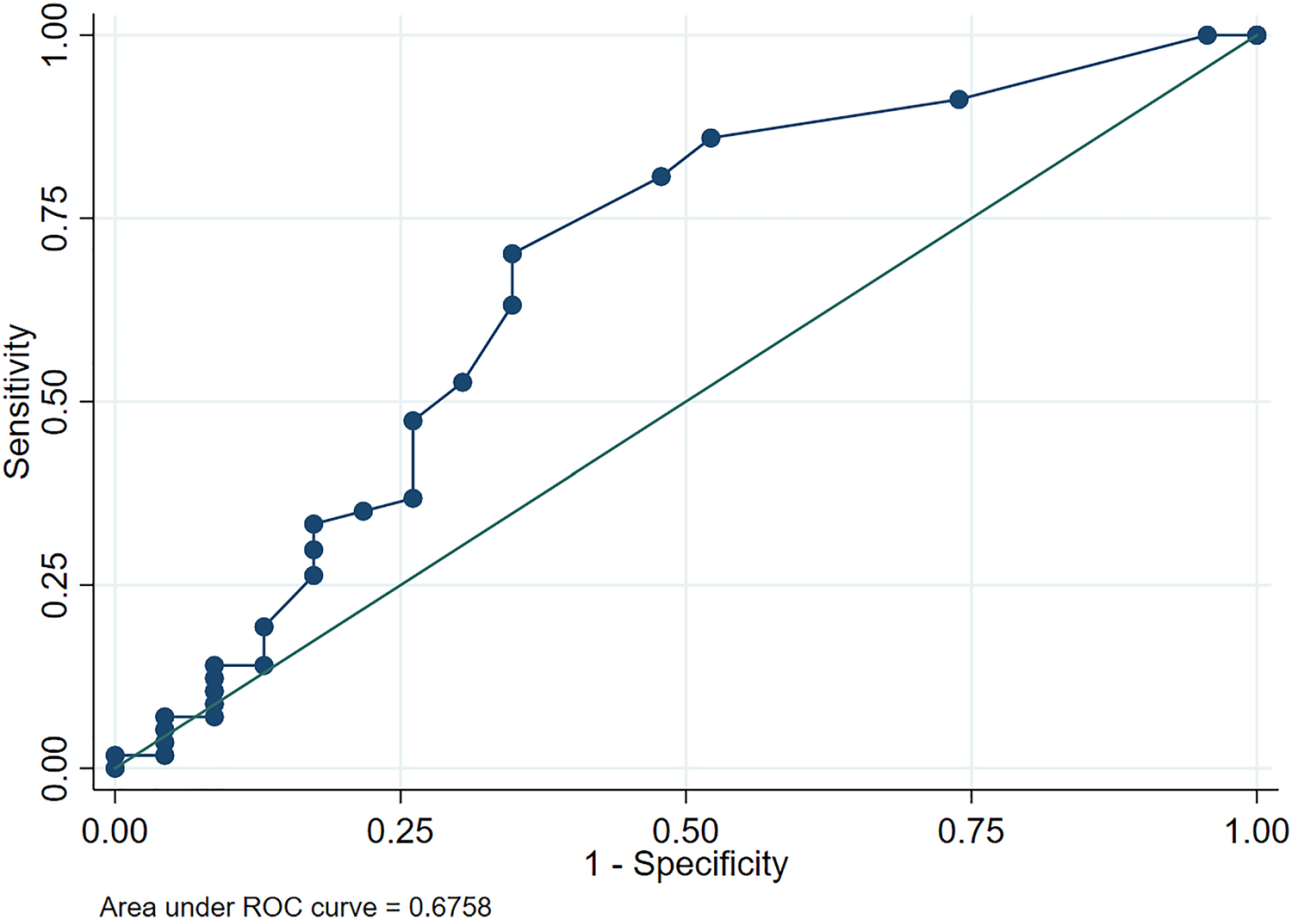
ROC curve of capillary ketonemia for the discrimination between NSAP and PAA

Regarding the capacity of CK to discriminate between patients from groups 1 and 2, we found an AUC of 0.68 (95% CI 0.53–0.82) (*p* = 0.24) (Fig. 2). The cut-off point with the best Youden Index resulted in 0.4 mmol/L, with a sensitivity of 80.70% and a specificity of 52.17%.

Table 4 summarizes the alternative proposed cut-off points with their corresponding values of sensitivity and specificity.

**Table 4:** Alternative cut-off points of capillary ketonemia for the discrimination between NSAP and PAA

Capillary ketonemia (mmol/L)	Positive likelihood ratio	Correctly classified (%)	Sensitivity (%)	Specificity (%)
0.4	1.68	72.50	80.70	52.17
0.7	1.72	57.50	52.63	69.57
1.2	1.92	47.50	33.33	82.61

### Diagnostic performance of CK to discriminate between complicated and uncomplicated PAA

**Fig. 3:**
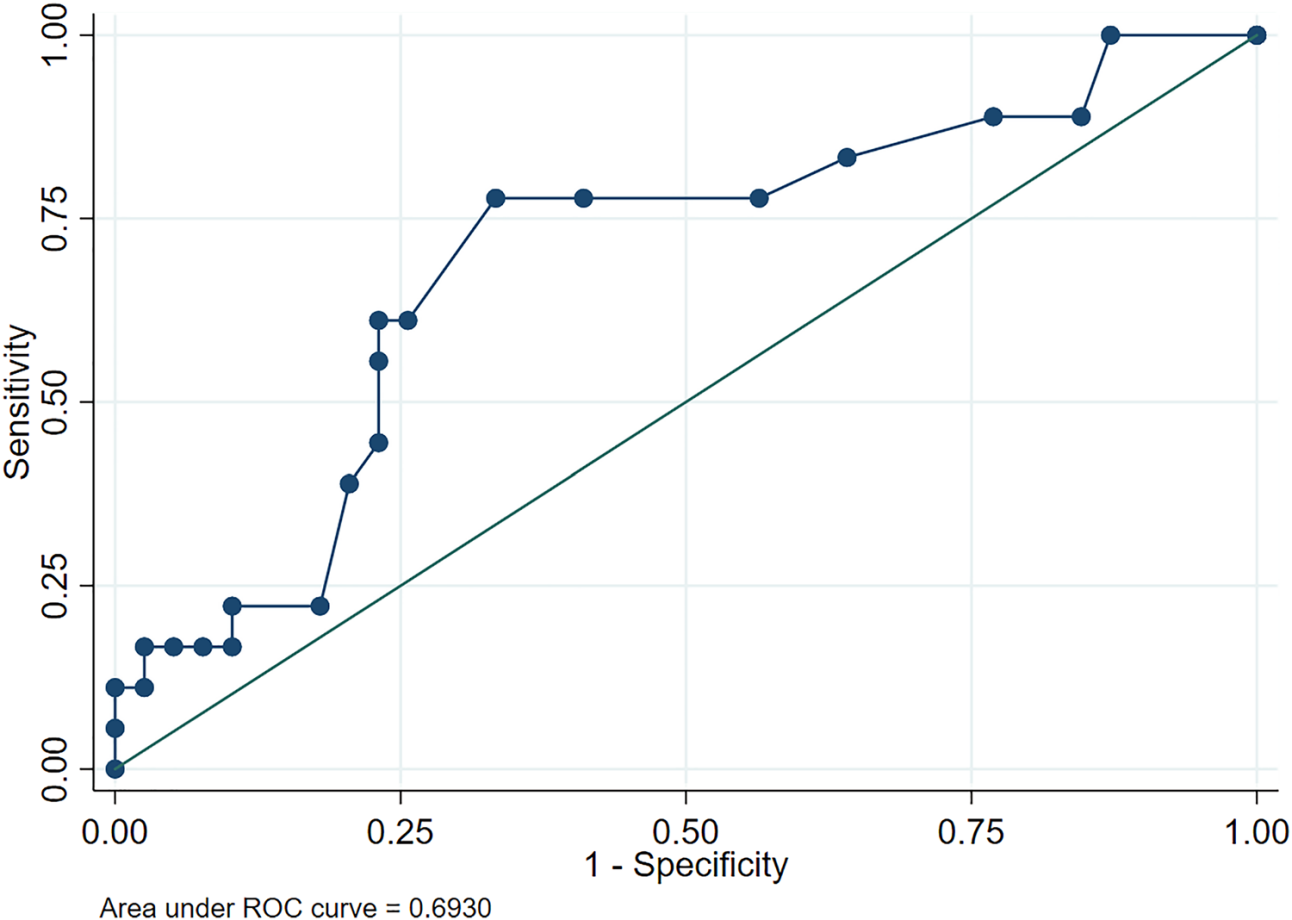
ROC curve of capillary ketone for the discrimination between complicated and uncomplicated PAA)

Regarding the capacity of CK to discriminate between complicated and uncomplicated PAA, we found an AUC of 0.69 (95% CI 0.54–0.85) (*p* = 0.04) (Fig. 3). The cut-off point with the best Youden Index resulted in 1.1 mmol/L, with a sensitivity of 61.1% and a specificity of 76.9%.

Table 5 summarizes the alternative proposed cut-off points with their corresponding values of sensitivity and specificity.

**Table 5:** Alternative cut-off points of capillary ketonemia for the discrimination between complicated and uncomplicated PAA

Capillary ketonemia (mmol/L)	Positive likelihood ratio	Correctly classified (%)	Sensitivity (%)	Specificity (%)
0.4	1.15	43.86	88.90	23.08
0.7	1.90	64.91	77.78	58.97
1.1	2.65	71.93	61.11	76.92
3.7	6.5	71.93	16.67	97.44

### Correlation analysis between CK and clinical and analytical variables

The results of the correlation analysis between CK and both clinical and analytical variables is summarized in Table 6. We found weak correlations between CK and total neutrophil count (*r* = 0.22), number of emetic episodes (*r* = 0.25), presence of nausea (rho = 0.25), hours of pain evolution (*r* = 0.24) and serum creatinine (*r* =  – 0.28). Moderate correlations were found between CK and presence of fever at home (rho = 0.44), axillary temperature at the ER (*r* = 0.33), INR (*r* = 0.37), serum level of sodium (*r* =  – 0.46), days of admission (rho = 0.32) and CRP level (*r* = 0.54). No significant correlations were found between CK and days of hospitalization or post-surgical complications.

**Table 6:** Correlation analysis between capillary ketonemia and both clinical and analytical variables

Variable	Pearson’s r/Spearman’s Rho	*p*-value
Number of emetic episodes	*r* = 0.25	0.03
Emergency room axillary temperature	*r* = 0.33	< 0.01
Hours of pain evolution	*r* = 0.24	0.03
Serum creatinine	*r* = – 0.28	0.01
Serum sodium	*r* = – 0.46	< 0.01
CRP	*r* = 0.54	< 0.01
INR	*r* = 0.37	< 0.01
Total neutrophil count	*r* = 0.22	0.05
Presence of fever at home	Rho = 0.44	< 0.01
Presence of nausea	Rho = 0.25	0.02
Days of admission	Rho = 0.32	0.02

## Discussion

This subanalysis of the BIDIAP cohort found that CK levels were significantly higher in patients with PAA than in those with NSAP, and also in patients with complicated than in those with uncomplicated PAA. The diagnostic yield of CK to distinguish between NSAP and PAA or between complicated and uncomplicated PAA was only moderate.

The etiopathogenic and pathophysiologic mechanisms underlying the elevation of CK in patients with PAA responds to multiple phenomena including an increased catabolism, emesis and fasting, all of them being independently associated with dehydration [8–10, 16]. On fasting, after the consumption of the glycogen reserves, lipolysis occurs, with the release of glycerol, which is transformed into glucose and free fatty acids, which are oxidized to ketone bodies. While in adults this process may take more than 24 h from the beginning of fasting, it is a much faster process in children, whose glycogen reserves last from 8 to 12 hours (4 hours in the case of infants). Moreover, children’s metabolism is faster than that of adults and therefore, children tend to present higher energy expenditure. All these factors condition an early onset of ketosis in the pediatric population during fasting [10, 16, 17].

In this study, we found that CK was directly correlated with the number of emetic episodes, the presence of fever at home, axillary temperature at the emergency department, and time of pain evolution. We believe these factors reflect an increase in catabolism and a gradual dehydration conditioned by fluid loss through the third space associated with acute abdomen, insensible losses associated with fever, and emesis.

In relation to the analytical parameters, we found inverse correlations between CK and both serum creatinine and sodium. The former being weak (r =  – 0.28) and the latter, moderate (r =  –0.46). Hyponatremia is a well-known alteration in complicated PAA, which agrees with our findings [18]. On the other hand, we expected the correlation between CK and creatinine to be positive. We hypothesize that these results may be due to the fact that catabolism and fasting have greater impact on children’s body than dehydration on their renal function.

We also found direct weak correlations between CK and total neutrophil count and INR, and a direct and moderate correlation between CK and CRP. This last finding could be attributable to two factors: the inflammation per se and the time of evolution.

First, the elevation of serum CRP usually translates a systemic inflammatory process, which, depending on its severity and exent, may have an impact on the degree of dehydration. Higher levels of CRP indicate greater extension of inflammation, which contributes to a higher risk of dehydration.

Second, serum levels of CRP are directly correlated with the time of evolution of several inflammatory pathologies, including appendicitis [19]. Therefore, higher levels of CRP usually reflect more advanced stages of those pathologies, which may conduct to a greater degree dehydration through the pathophysiological mechanisms previously mentioned (i.e fever, fasting, emesis).

Regarding the clinical utility of CK, it must be acknowledged that it is a widely available and inexpensive biomarker. In our study, the cost of CK determination resulted in less than 2 euros per patient. In addition, the result is obtained instantaneously, which, compared to other markers that require additional processing in the laboratory, may represent an enormous advantage in clinical practice.

Given that the main limitations for hospital discharge in patients operated on PAA are postoperative emesis and hyporexia, we consider the results of our study could be of great interest. We believe that it would be possible to develop protocols for pediatric emergency departments with routine measurement of CK and subsequent application of a rehydration regimen according to the result obtained prior to surgery. We believe that this type of protocol could serve to accelerate the recovery of patients operated on PAA, thus reducing the stay at hospital.

This study has several strengths, including the prospective design, the large sample size and the thorough statistical analyses. Despite our findings, we must acknowledge some limitations. First, we used a convenience sampling, which is susceptible of a selection bias. Besides, 71 patients were excluded due to missing data. This could lead to selection bias, so an exhaustive comparative analysis was performed between the two subpopulations of the cohort (patients with CK determination and patients without determination) for all sociodemographic and clinical variables, and no statistically significant differences were found in any of the analyses. However, the possibility of residual confounding secondary to statistically significant differences in parameters not specifically assessed in this work should be considered. As authors, however, we believe that the strict application of inclusion and exclusion criteria that we followed makes this a low risk.

In conclusion, patients with PAA have significantly higher levels of CK than patients with NSAP. Similarly, patients with complicated PAA have significantly higher levels of CK than patients with uncomplicated PAA. Nevertheless, the diagnostic performance of this molecule in the setting of PAA is only moderate. The potential usefulness of CK determination as a tool to guide the preoperative rehydration regimen of patients with PAA to prevent postoperative hyporexia and vomiting is a promising line of research and should be evaluated in future studies.

### Supplementary Information

Below is the link to the electronic supplementary material.

Supplementary file1 (DOCX 15 KB)

## Data Availability

All data pertaining to this study are available upon justified request through the author in correspondence.
